# New time-scale criteria for model simplification of bio-reaction systems

**DOI:** 10.1186/1471-2105-9-338

**Published:** 2008-08-12

**Authors:** Junwon Choi, Kyung-won Yang, Tai-yong Lee, Sang Yup Lee

**Affiliations:** 1 Department of Chemical and Biomolecular Engineering (BK21 Program), KAIST, 335 Gwahangro, Yuseong-gu, Daejeon, 305-701, Republic of Korea; 2 Center for Systems and Synthetic Biotechnology, Institute for the BioCentury, KAIST, 335 Gwahangro, Yuseong-gu, Daejeon, 305-701, Republic of Korea

## Abstract

**Background:**

Quasi-steady state approximation (QSSA) based on time-scale analysis is known to be an effective method for simplifying metabolic reaction system, but the conventional analysis becomes time-consuming and tedious when the system is large. Although there are automatic methods, they are based on eigenvalue calculations of the Jacobian matrix and on linear transformations, which have a high computation cost. A more efficient estimation approach is necessary for complex systems.

**Results:**

This work derived new time-scale factor by focusing on the problem structure. By mathematically reasoning the balancing behavior of fast species, new time-scale criteria were derived with a simple expression that uses the Jacobian matrix directly. The algorithm requires no linear transformation or decomposition of the Jacobian matrix, which has been an essential part for previous automatic time-scaling methods. Furthermore, the proposed scale factor is estimated locally. Therefore, an iterative procedure was also developed to find the possible multiple boundary layers and to derive an appropriate reduced model.

**Conclusion:**

By successive calculation of the newly derived time-scale criteria, it was possible to detect multiple boundary layers of full ordinary differential equation (ODE) models. Besides, the iterative procedure could derive the appropriate reduced differential algebraic equation (DAE) model with consistent initial values, which was tested with simple examples and a practical example.

## Background

The dynamic simulation of bio-reaction pathways is becoming more important as the kinetic information of various pathways is revealed. Moreover, the necessary data for the specific pathways are easily obtained through various channels, including the internet. However, there are fundamental difficulties in the numerical solution of the differential equation system: model stiffness is one. a bio-reaction system consists of multiple reactions with various enzymes that have different turn-over numbers, meaning various magnitudes of reaction rates. Furthermore, the metabolites involved in one reaction can also participate in other reactions in the same system. These characteristics make typical bio-systems strongly coupled and have multiple time-scales. Therefore, ordinary differential equations (ODEs) based on the dynamic modeling of a metabolic system are usually stiff.

The stiffness problem requires unnecessary effort to track the boundary layer solutions, hence, the computational efficiency decreases. Furthermore, when the simulation is concurrent with the experiment, the calculation accuracy is closely related to the measurement interval. If the measurement interval is modest so that it is impossible to find some specific parameters, then the numerical result does not need to be precise. In these situations, by sacrificing the accuracy modestly, simplifying the model structure is necessary, which is often the case for parameter estimations and sensitivity analyses [1].

For this purpose, apart from a numerical analysis approach, the kinetic field's specific solution methods have been required [2]. Traditionally, simplification of an original complex model, such as a quasi-steady state approximation (QSSA) and a partial equilibrium approximation, have been applied to relieve the stiffness characteristics [3-7]. However, since these approaches require the practitioner's intuition and experience, computational methods have been developed, especially vividly in combustion engineering fields. There are two important procedures in computational model simplification: the determination of the simplification criteria and the determination of the slow invariant manifolds.

Some computational methods concentrated on deriving the correct slow manifolds. They are iterative trajectory based methods [8,9], the method of invariant manifold (MIM) [10], the minimal entropy production trajectory (MEPT) based methods [11,12], and the nonlinear model reduction method [13]. Gorban *et al*. collected and reviewed such kinds of methods [14].

The others suggested the appropriate simplification criteria: generalized sensitivity analyses [15] used singular values of the sensitivity matrix as the scale factor, while computational singular perturbation (CSP) [16-18], intrinsic low dimensional manifold (ILDM) [19], and dynamic dimension reduction, which is a modified version of ILDM [20,21], used the eigenvalue analysis of the system's Jacobian matrix with focusing on the time-scales of the system. Currently, ILDM based methods have been applied to reduce complexity of biochemical systems [21-23]. Since CSP or ILDM based methods use the dynamic properties of a system, they can give the dynamically useful information of the system.

Even if there are several differences between the CSP and ILDM, the most important ideas are similar: the determination of the speed ranking is based on the eigenvalue calculation of the Jacobian matrix, which require at least *O*(*n*^3^) flops of computation, and the derivation of solution is based on a linear transformation of the original system. Furthermore, they share common barriers in producing an explicitly reduced model, generally as a result of the system transformation. This feature is important when model simplification approaches are related to not only computational efficiency but also parameter estimation.

This work suggests an automatic method for speed ranking that directly uses the Jacobian matrix without a system transformation. Due to its simplicity, this approach requires *O*(*n*) flops of computation, being more physically intuitive. In addition to the scaling procedure, the decision of the differential and algebraic variables in a slow dynamic regime after relaxation of the fast dynamics is also introduced. The result of the proposed process is an explicitly phrased model, which can be a route to distinguish the meaningful parameters from the unobservable ones.

## Methods

### Criteria for balancing

In homogeneous chemical kinetics, the dynamic model can be written in the following form of the ODE.

dyidt=fi(y)=∑j=1msijvji=1,...,n,0≤t≤tf.

Since a chemical reaction system generally consists of production and loss terms, the ODE can be rewritten as:

(1) dyidt=Pi(y)−Li(y)=∑sP,ijvj−∑sL,ijvjsP,ij=max⁡(sij,0)sL,ij=sP,ij−sij,

or with a matrix-vector notation,

(2) y˙=S v(y)=SP v−SL v,

where **y **∈ ℝ^+*n *^is a concentration vector, *P*_*i*_: ℝ^+*n *^→ ℝ^+ ^is a production term, and *L*_*i *_: ℝ^+*n *^→ ℝ^+ ^is a loss terms. **S **is a stoichiometric matrix and **v **is a reaction rate vector. The subscripts *P *and *L *denote the production and loss, respectively.

Generally, it can be said that if *y*_*i *_exhibits a quasi-steady state behavior, such behavior is observed after a short period of time for the corresponding *P*_*i *_and *L*_*i *_to balance each other. In a normal computational environment, the period can be readily estimated from equation (1), but the applicability of QSSA cannot be directly determined based on the estimation. The goal of this work is to determine the proper criteria to determine the applicability of the QSSA based on the estimation of balancing period. At the moment either *P*_*i *_or *L*_*i *_enlarge, the period during which *P*_*i *_balances with *L*_*i *_can be evaluated in a simple manner.

By chain rule,

$$\begin{array}{c} \frac{dP_{i}}{dt}={\left(\frac{\partial P_{i}}{\partial y}\right)}^{T}f\\ \frac{dL_{i}}{dt}={\left(\frac{\partial L_{i}}{\partial y}\right)}^{T}f, \end{array}$$

where **f **= (*f*_1_, *f*_2_, . . .)^*T*^. Let *δt*_*i *_be a short time period after which *P*_*i *_and *L*_*i *_balance each other. Then, we have following relationship:

$$L_{i}^{0}+{\frac{dL_{i}}{dt}|}_{y^{0}}\delta t_{i}\approx P_{i}^{0}+{\frac{dP_{i}}{dt}|}_{y^{0}}\delta t_{i},$$

where superscript 0 indicates the reference value. Rearranging the equation gives:

(3) $$\delta t_{i}\approx \frac{L_{i}^{0}-P_{i}^{0}}{{\frac{dP_{i}}{dt}|}_{y^{0}}-{\frac{dL_{i}}{dt}|}_{y^{0}}}.$$

If the denominator on the righthand side of equation (3) is not zero, we can compute the time scale *δt*_*i *_from this equation. If the magnitude of the time scale is large, namely, |*δt*_*i*_| > *ϵ*_*t *_for some *ϵ*_*t *_> 0, *y*_*i *_is considered to exhibit slow dynamics and QSSA is not applied.

If |*δt*_*i*_| <*ϵ*_*t*_, *y*_*i *_exhibits fast dynamics, QSSA can be applied depending on the sign of *δt*_*i*_.

• If *δt*_*i *_is positive, it will reach a balancing state quickly and QSSA can be applied to *y*_*i*_.

• If *δt*_*i *_is negative, the applicability of QSSA cannot be determined by *δt*_*i *_alone.

If the denominator is zero, the numerator *L*_*i *_- *P*_*i *_should be considered for following three cases:

• *P*_*i *_≠ *L*_*i *_indicates a non-reducing state between production and loss. QSSA should not be applied.

• *P*_*i *_= *L*_*i *_= 0 indicates that no dynamics has occurred yet for *y*_*i*_. QSSA should not be applied.

• *P*_*i *_= *L*_*i *_≠ 0 indicates that *δt*_*i *_should be set to zero since complete balance has been obtained.

The time scale of the element reaction can be also estimated using the same method, which will be used to determine a closed subsystem later.

(4) $$\delta \tau_{j}\approx -{\left(\frac{d\ln v_{j}^{0}}{dt}\right)}^{-1}.$$

Another scale factor should be used to determine whether the *i*'th variable is balanced or not; the ratio of |*f*_*i*_| to the larger one of *P*_*i *_and |*L*_*i*_|,

(5) $$r_{i}=\frac{\left|f_{i}\right|}{\max(P_{i},L_{i})}.$$

If -*ϵ*_*t *_= *δt*_*i *_< 0, the information about *r*_*i *_becomes important to determine the dynamics of *y*_*i*_. If *r*_*i *_is large, namely *r*_*i *_> *ϵ*_*r *_for some *ϵ*_*r *_> 0, the production and loss are neither balanced nor can be balanced soon, hence QSSA is not applicable to such *y*_*i*_.

Based on the values calculated from (3) to (5), the criteria to separate QSSA variables are as follows.

1. Both production and loss terms must exist.

2. For certain *ϵ*_*r *_≪ 1 and *ϵ*_*t *_≪ *t*_*f*_, the applicability of QSSA to the *i*th variable can be summarized as in Table 1.

**Table 1:** QSSA applicability.

	0 ≤ *δt*_*i *_≤ *ϵ*_*t*_	-*ϵ*_*t *_<*δt*_*i *_≤ 0	|*δt*_*i*_| > *| ϵ *_*t*_
*r*_*i *_≤ *ϵ*_*r*_	Yes	Yes	No
*r*_*i *_> *ϵ*_*r*_	Yes	No	No

Applicability of QSSA to *y*_*i*_

### Iterative balancing

The above-mentioned scale factors *δt*_*i *_and *r*_*i *_are locally determined at a certain moment, initially in the innermost boundary layer. Therefore, if there are multiple boundary layers, the separation process should be applied iteratively to exit the fastest dynamic regime and move to the next fastest one, jumping to the next boundary layers, and finally to the slow dynamic regime, outer region, where fast dynamics of the inner regions are fully relaxed. The iterative procedure locates the variable values of the fast dynamics, **y**(0), to the outer region, **y**(0^+^), which become the consistent initial values of the reduced system.

Once *δt*_*i *_and *r*_*i *_are computed, identifying the fast variables, one can decompose the original stoichiometric matrix into two sub-matrices, **S**_*f *_and **S**_*s*_. Equation (2) is rewritten as:

y˙s=Ssvy˙f=Sfvy=(ysyf),

where subscript *s *indicates *slow *variables and *f *indicates *fast *ones. To derive consistent initial values for the outer region, the following algebraic equation should be solved:

(6) 0 = **S**_*f *_**v**.

The solution of equation (6) can give other scale factors that also satisfy the approximation criteria, which shows the possibility of multiple boundary layers with different time scales. The existence of multiple boundary layers with different time scales corresponds to the cascaded nested hierarchy concept of inertial manifolds [8]. For these events, equations for the iterative approach can be written as:

(7) $$0=S_{y}^{k}v(y_{f}^{k}),$$

where the superscript *k *is the number of iterations. From $y_{f}^{k}$, $\delta t_{i}^{k}$ and $r_{i}^{k}$ are iteratively calculated and the updated equation (7) is solved until |**y**^*k*+1 ^- **y**^*k*^| satisfies the convergence criteria. The convergence criterion of this iterative procedure can be considered as the partial equilibrium among the fast variables. This is conceptually similar to the equilibrium value convergence of Lebiedz's work [11]. After convergence is achieved, the following reduced models with the modified initial values are derived:

(8) y˙s=Ssv0≈Sfv,y0=y(0+).

The solution of the algebraic equation (7) can be computed using the appropriate numerical method such as Newton's method or other similar methods. However, those methods sometimes give physically meaningless solutions. To overcome this, Mass and Pope applied an iterative technique that successively constructs sub-ODE systems and successively solves them [19]. This work applied a similar approach to that of Mass and Pope to find the plausible initial guesses for the algebraic equation (7).

From equation (7), the columns of **S**_*f*_, say **s**_*j*_, give information about the closing and opening of the subsystem by the *j *'th reaction. If **s**_*j *_is composed of only non-negative signs or only non-positive signs, this system is opened by the *j*'th reaction. If the time scale of the opening *j*'th reaction, *δτ*_*j*_, is large, say *δτ*_*j *_> *ϵ*_*t*_, the reaction is excluded from the subsystem, resulting in a closed subsystem,

(9) $${\dot{y}}_{f}^{k}=S_{fc}^{k}v.$$

Since the closed system always has steady state, the *k*'th solution of the transient subsystem reaching a steady state is used as the initial guess for equation (7).

### Overall process

The criteria are used to detect the existence of a boundary layer and the iterative balancing computes the initial values of the reduced model at the border of the outer region. There are two categories of QSSA possibilities. The first category is that the large deviation between *P*_*i *_and *L*_*i *_decreases very fast, which gives a relatively larger *r*_*i *_but a small *δt*_*i*_. The second is that *P*_*i *_and *L*_*i *_are almost balanced by a coincidental initial value. In this case, the approximation is also dependent on how small *δt*_*i *_is. Then the iterative balancing relocates the initial values toward the outer region. If there are multiple boundary layers, a few more iterations of the iterative balancing are required to search for the proper initial values for the reduced model. Once the iterative balancing converges, indicating every possible balancing is completed with small values for both of *r*_*i *_and *δt*_*i*_, it is assumed that the fast dynamics are relaxed and slow dynamics of the outer region begin with the updated initial values.

This process can also identify the boundary layers that occur internally, not only at the initial point, because the detection checks the possibility of QSSA at every step of the calculation. These features are illustrated in the next section. In summary, the overall detecting and balancing process is as follows:

### Iterative balancing process

1. Calculate *δt*_*i*_, *δτ*_*j*_, and *r*_*i *_using equations (3) through (5).

2. If no species can be approximated, then go to step 4, or else go to step 3.

3. Perform iterative balancing using equation (7).

4. Solve the ODE/DAE model with equation (8).

5. Repeat steps 1 to 4 until the current *t *reaches the user-defined *t*_*f*_.

## Results and Discussion

**Figure 1:**
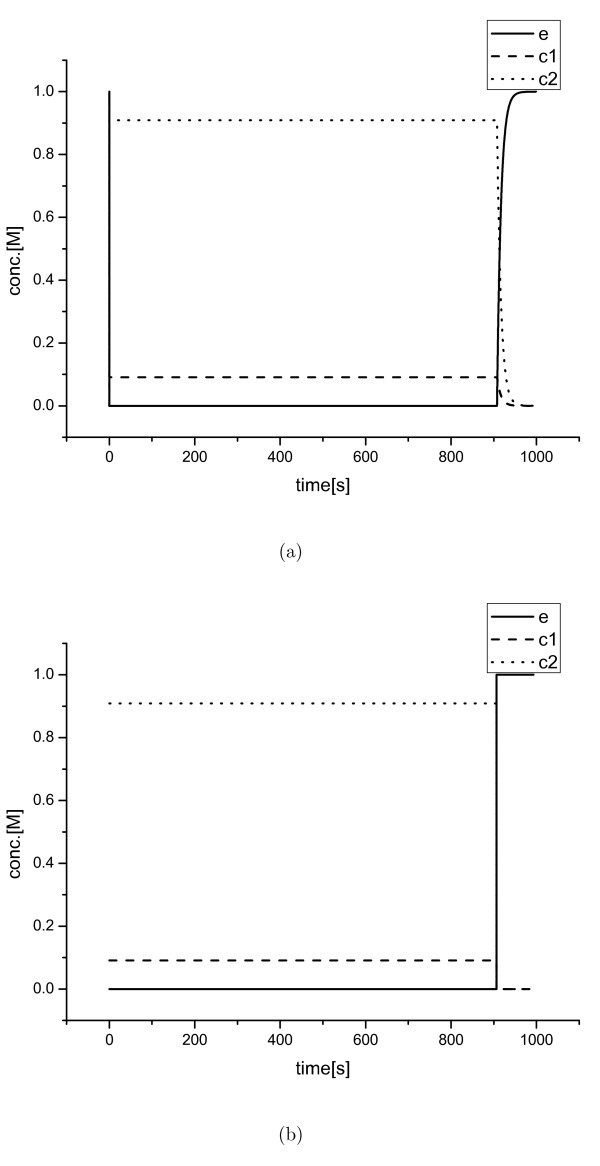
**Simplification results (I)**. Concentrations of *e*, *c*_1_, and *c*_2 _for the Michaelis-Menten system; (a) full ODE model solution and (b) reduced model solution.

**Figure 2:**
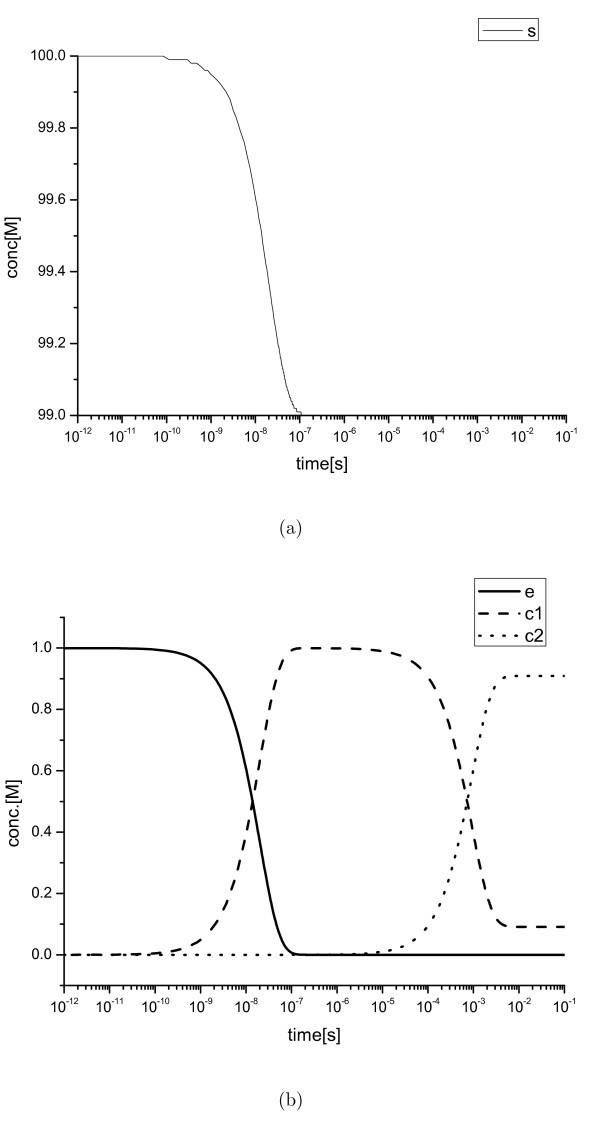
**Existence of multiple boundary layers**. (a) Semi-log plot of *s *and (b) that of *e*, *c*_1_, and *c*_2 _for the Michaelis-Menten system. The existence of two boundary layers at the initial region are observed.

The Michaelis-Menten kinetics without inhibition,

(10) $$e+s\overset{k_{1}}{\underset{k_{2}}{⇌}}c_{1}\overset{k_{3}}{\underset{k_{4}}{⇌}}c_{2}\overset{k_{5}}{\underset{}{\to }}p+e,$$

and with inhibition,

(11) $$\begin{array}{lll} e+s & \overset{k_{1}}{\underset{k_{2}}{⇌}} & es_{1}\overset{k_{3}}{\underset{k_{4}}{⇌}}es_{2}\overset{k_{5}}{\underset{}{\to }}p+e\\ e+i & \overset{k_{1}}{\underset{k_{2}}{⇌}} & ei, \end{array}$$

are considered in this study. The parameters are (*k*_1_, *k*_2_, *k*_3_, *k*_4_, *k*_5_) = (500000, 5, 1000, 100, 0.16) and the initial values are (*e*_0_, *s*_0_, *c*_10_, *c*_20_, *p*_0_) = (1, 100, 0, 0, 0) and (*e*_0_, *s*_0_, *c*_10_, *c*_20_, *p*_0_, *i*_0_, *ei*_0_) = (1, 100, 0, 0, 0, 1000, 0) [24]. As can be seen, there are two boundary layers at the initial region and near *t *= 900 (see Figure 1). Since *p *is only produced, its dynamics are not considered when searching for the fast balancing species. In the initial region, the estimated value of *δt *≈ 3.96 × 10^-8 ^and species *e, s*, and *c*_1 _were selected as fast variables, as expected. The subsystem composed of the chosen species was opened by the second reaction, hence the second reaction was removed from the subsystem. Based on the solution from the first iteration, species *c*_1 _and *c*_2 _were selected. Since the *δt *of *e *and *s *remains small, there indices were maintained as fast variables. The updated subsystem was opened by the third reaction, and consequently, the reaction was excluded. After the second iteration, the solution converged and was stored, then the open system was solved in a small interval period. Since a very large *δt *of *s *was identified in this refining step, *s *was excluded from the fast variable set. Finally, *e*, *c*_1_, and *c*_2 _were selected as QSSA variables before the second boundary layer. The values of *δt*_*i *_at each iteration after three iterations of the iterative process are listed in Table 2. At the second boundary layer, the predefined criteria gave another iterative process and relocated the solution toward the outer area in the same manner as described above. For comparison with the conventional manual QSSA approach, the time scales of each species were also derived by mathematical balancing [25]. The meaning of the time-scales of the fast variables from the conventional derivation is the time to exit the boundary layer. Therefore, the summation of *δt*_*i *_for every iteration until the species enters the outer region is the direct comparative value of the conventional scales (see Table 3). A similar tendency is observed between the sums of *δt*_*i *_and the mathematical scales. As in Table 3, the sums of *δt*_*i *_and the mathematically scaled values indicate that there are two boundary layers near *t *≈ 10^-8 ^and *t *≈ 10^-3 ^before exiting the initial regions. A semi-log plot of the full model simulation (Figure 2) supports this expectation.

**Table 2:** *δt *values.

Iteration	0	1	2
*e*	3.96 × 10^-8^	-3.09 × 10^-13^	3.57 × 10^-13^
*s*	3.96 × 10^-8^	-3.09 × 10^-13^	3.51 × 10^2^
*c*_1_	3.96 × 10^-8^	1.81 × 10^-3^	4.57 × 10^-11^
*c*_2_	Not available	1.82 × 10^-3^	1.82 × 10^-3^

*δt *values during the initial iterative process of two complex Michaelis-Menten kinetics

**Table 3:** Comparison of time-scales.

	Sums of *δt*	Mathematical scale
*e*	3.96 × 10^-8^	2.00 × 10^-8^
*s*	3.51 × 10^2^	6.25 × 10^2^
*c*_1_	1.81 × 10^-3^	1.00 × 10^-3^
*c*_2_	0.36 × 10^-2^	1.00 × 10^-2^

Mathematically derived time scales and sums of *δt*_*i *_of two complex Michaelis-Menten kinetics

**Figure 3:**
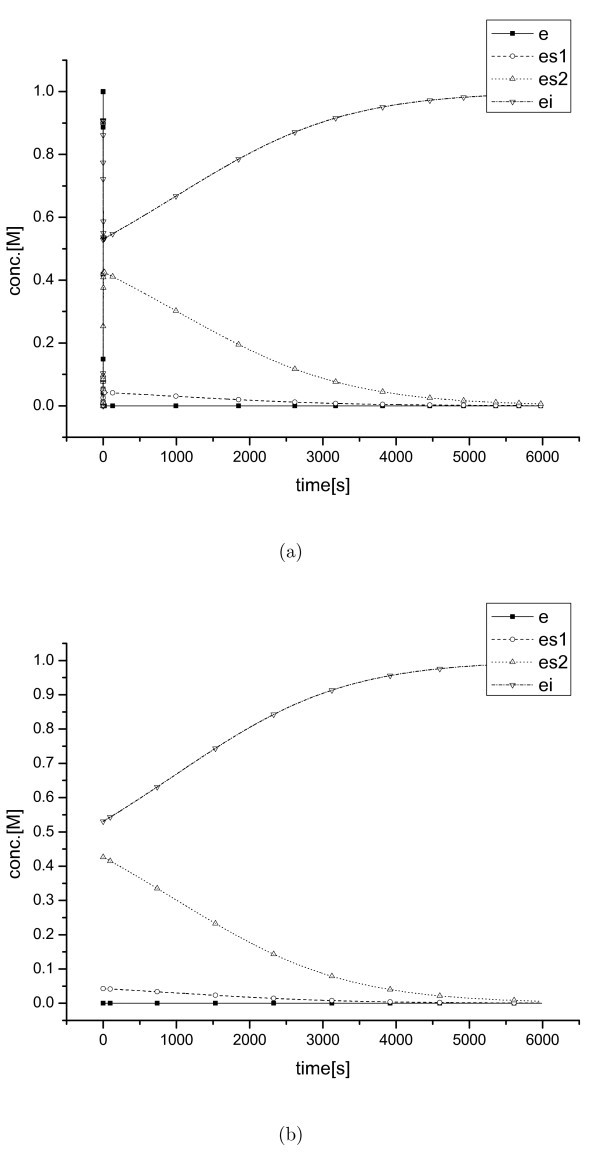
**Simplification results (II)**. Concentrations of *e*, *es*_1_, *es*_2_, and *ei *for the Michaelis-Menten system with inhibition; (a) full ODE model solution and (b) reduced model solution.

The second example, the Michaelis-Menten kinetics with inhibition, shows a boundary layer at the initial area only (see equation (11) and Figure 3) with a similar scale of *δt *to that of the non-inhibition case. The dynamic behavior of the second model in the inner region of the initial boundary layer is more complex because of the effect of the inhibition. These complex dynamics of the second example require a few more iterations than that of the first example to exit the initial boundary layer.

The third example is the caspase activation model given by equation (12) in [26].

(12) d[c8]dt=−v2−v9d[c8∗]dt=v2−v5−v11d[c3]dt=−v1−v10d[c3∗]dt=v1−v3−v6d[IAP]dt=−v3−v4−v8d[c3∗~IAP]dt=v3−v7d[BAR]dt=−v11−v12d[c8∗~BAR]dt=v11−v13

**Figure 4:**
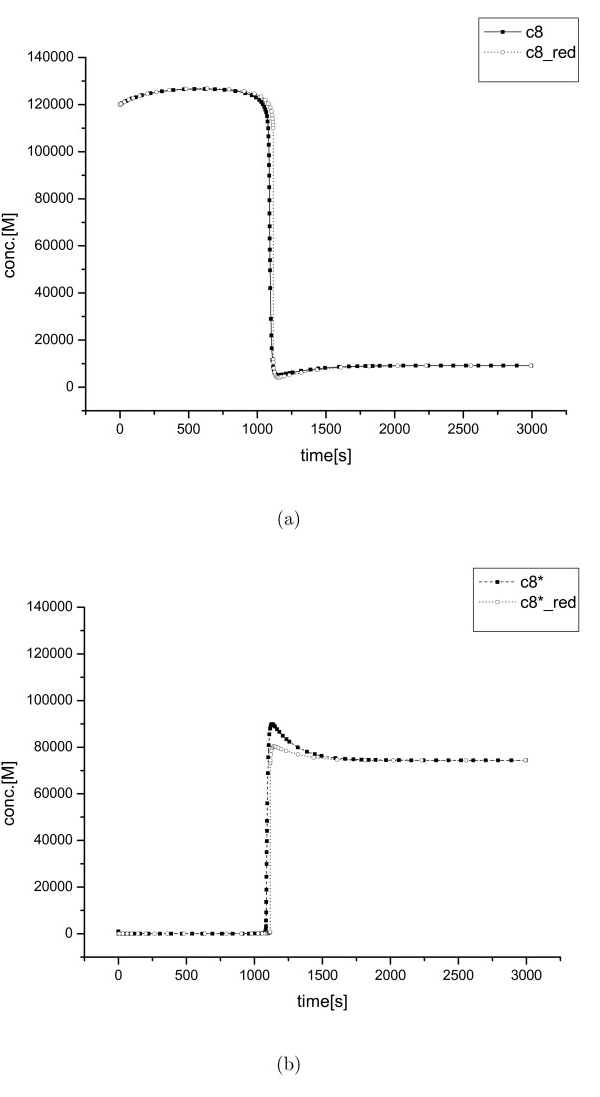
**Simplification results (III)**. Concentrations of c8 and c8* for the caspase system; (a) the solution profile of c8, from the full ODE model (c8) and from the reduced model (c8_red) and (b) the solution profile of c8*, from the full ODE model (c8*) and from the reduced model (c8*_red).

The reaction rate equations for equation (12) are written as *v*_1 _= *k*_1_[c8*][c3], *v*_2 _= *k*_2_[c3*][c8], *v*_3 _= *k*_3_[c3*][IAP] *- k*_-3 _[c3*~IAP], *v*_4 _= *k*_4_[c3*][IAP], *v*_5 _= *k*_5_[c8*], *v*_6 _= *k*_6_[c3*], *v*_7 _= *k*_7_[c3*~IAP], *v*_8 _= *k*_8_[IAP] - *k*_-8_, *v*_9 _= *k*_9_[C8] - *k*_-9_, *v*_10 _= *k*_10_[c3] - *k*_-10_, *v*_11 _= *k*_11_[c8*][BAR] - *k*_-11 _[c8*~BAR], *v*_12 _= *k*_12_[BAR] - *k*_-12 _and *v*_13 _= *k*_13_[c8a~BAR], where the kinetic constants are listed in [26]. There are also two boundary layers at the initial and internal regions, but with a much larger *δt *relative to the former cases; *δt *≈ *O*(10^-1^) at the initial area and *δt *≈ *O*(1) at the internal boundary layer (Figure 4).

Although it is difficult to recognize the initial boundary layer in the figure because of the small change in the concentrations relative to that of the internal boundary layer, the initial boundary layer exists in the third model, which can be overlooked by the simple observation of the full model simulation. The existence of the initial boundary layer of the third model could be detected by the proposed algorithm.

Figure 1 and Figure 3 show that the exact value of the parameters *k*_1_, *k*_2_, *k*_3_, and *k*_4 _cannot be properly identified using measurement intervals larger than *O*(10^-8^). Figure 4 also illustrates that it is impossible to obtained the specific parameters of the original model with a measurement interval larger than *O*(1). Only the ratios of some species's concentrations can be observed. Therefore, the ratios are meaningful for these situations, not the detailed dynamics occurred within the period smaller than the measurement interval. The suggested scheme improves computational efficiency in the stiff inner region and extracts the information of experimentally meaningful QSSA concentrations for the corresponding species. Besides, by determining a measurement scale, the level of simplification can be controlled easily.

## Conclusion

This work proposed new criteria for the time-scale analysis and iterative balancing approach to develop an automatic simplification. This approach has different consistent initial values after model reduction and successfully found consistent initial values of the simplified DAE model using iterative balancing. With some examples, from small systems to practical systems, this scheme gave a successful reduction and found consistent initial values. If a whole cell is the system of the dynamic simulation, the network of reaction pathways will be more complex, the number of variables will be increased, and the simulation will be more difficult. Henceforth, it may be also important to relate the derived model to the experimental view, and this approach can give the criteria to classify the meaningful values from the original model.

## Authors' contributions

JC developed and implemented the algorithm and prepared the manuscript. K–WY implemented the subroutines of the proposed algorithm. T–YL corrected the mathematical expression and participated in the manuscript preparation. SYL participated in the design of the study. All authors read and approved the final manuscript.
